# Early ART After Cryptococcal Meningitis Is Associated With Cerebrospinal Fluid Pleocytosis and Macrophage Activation in a Multisite Randomized Trial

**DOI:** 10.1093/infdis/jiv067

**Published:** 2015-02-04

**Authors:** James E. Scriven, Joshua Rhein, Katherine Huppler Hullsiek, Maximilian von Hohenberg, Grace Linder, Melissa A. Rolfes, Darlisha A. Williams, Kabanda Taseera, David B. Meya, Graeme Meintjes, David R. Boulware

**Affiliations:** 1 Infectious Diseases Unit, GF Jooste Hospital, Cape Town; 2 Clinical Infectious Diseases Research Initiative, Institute of Infectious Disease and Molecular Medicine, University of Cape Town, South Africa; 3 Liverpool School of Tropical Medicine, United Kingdom; 4 Division of Infectious Diseases and International Medicine, Department of Medicine, University of Minnesota, Minneapolis; 5 Infectious Disease Institute, Makerere University, Kampala, Uganda; 6 Division of Biostatistics, School of Public Health, University of Minnesota, Minneapolis; 7 Mbarara University of Science and Technology; 8 Department of Medicine, School of Medicine, College of Health Sciences, Makerere University, Kampala, Uganda; 9 Department of Medicine, Faculty of Health Sciences, University of Cape Town, South Africa; 10 Department of Medicine, Imperial College London, United Kingdom

**Keywords:** AIDS, cryptococcal meningitis, HIV, immunology, IRIS, macrophage, randomized controlled trial, sCD14, sCD163

## Abstract

***Introduction.*** Earlier antiretroviral therapy (ART) initiation in cryptococcal meningitis resulted in higher mortality compared with deferred ART initiation (1–2 weeks vs 5 weeks postmeningitis diagnosis). We hypothesized this was due to ART-associated immune pathology, without clinically recognized immune reconstitution inflammatory syndrome.

***Methods.*** Three macrophage activation markers and 19 cytokines/chemokines were measured from cryopreserved cerebrospinal fluid (CSF) and serum during the Cryptococcal Optimal ART Timing (COAT) trial. Comparisons were made between trial arms (early vs deferred) at 1, 8, 14, and 21 days following meningitis diagnosis.

***Results.*** More participants with early ART initiation had CSF white cell count (WCC) ≥5/µL at day 14 (58% vs 40%; *P* = .047), after a median of 6-days ART. Differences were mainly driven by participants with CSF WCC <5/µL at meningitis diagnosis: 28% (10/36) of such persons in the early ART group had CSF WCC ≥5/µL by day 14, compared with 0% (0/27) in the deferred arm (*P* = .002). Furthermore, Kampala participants (the largest site) receiving early ART had higher day-14 CSF levels of interleukin-13 (*P* = .04), sCD14 (*P* = .04), sCD163 (*P* = .02), and CCL3/MIP-1α (*P* = .02), suggesting increased macrophage/microglial activation.

***Conclusions.*** Early ART initiation in cryptococcal meningitis increased CSF cellular infiltrate, macrophage/microglial activation, and T helper 2 responses within the central nervous system. This suggests that increased mortality from early ART in the COAT trial was immunologically mediated.

Despite increasing availability of antiretroviral therapy (ART), cryptococcal meningitis (CM) remains a significant cause of death among individuals with human immunodeficiency virus (HIV)-1 infection in sub–Saharan Africa [1–3]. In a pooled study of 501 individuals with cryptococcal meningitis in South Africa, Malawi, Uganda, and Thailand, mortality was 17% at 2 weeks and 34% at 10 weeks [4]. In routine care, outcomes are substantially worse [5]. Efforts to improve survival with early ART initiation, a beneficial strategy with other opportunistic infections, including tuberculosis [6–8], have been unsuccessful in cryptococcosis. Two clinical trials conducted in sub–Saharan Africa have now been halted prematurely due to increased mortality with earlier ART initiation. The first, conducted in Zimbabwe using fluconazole 800 mg/day as induction antifungal therapy, found ART initiation within 72 hours of diagnosis led to a 3-fold increase in mortality compared with ART initiation at 10 weeks [9]. Recently, the COAT (Cryptococcal Optimal ART Timing) trial, conducted in Uganda and South Africa using amphotericin 0.7–1 mg/kg/day and fluconazole 800 mg/day as induction therapy, found a 15% increase in all-cause mortality when ART was initiated 1–2 weeks after diagnosis compared with deferred initiation at 5 weeks (26-week mortality, 45% vs 30%, respectively) [10].

The reasons for this increased mortality following early ART initiation remain unclear. In both trials, the majority of the deaths occurred within the first 4 weeks following ART initiation and was largely attributed to cryptococcosis. In the Zimbabwean trial, immune reconstitution inflammatory syndrome (IRIS) was not ascertained as an endpoint; in the COAT trial, earlier ART was associated with a nonstatistical increased incidence of IRIS. Mortality in the COAT trial particularly differed among individuals with cerebrospinal fluid (CSF) white cell counts (WCCs) <5 cells/µL at time of randomization, with earlier ART being detrimental [10]. Paucity of CSF immune responses during CM has been identified as a major risk factor for cryptococcal-IRIS [11, 12]. This raises the possibility that an inflammatory mechanism related to early ART caused the excess mortality observed in these 2 trials, resulting in sudden death rather than evolving to what was clinically recognized as paradoxical IRIS.

We hypothesized that earlier ART initiation resulted in excess inflammation in persons treated for CM. To test this, we compared the immune response of COAT trial participants, in CSF and serum, between the 2 trial arms (early vs deferred ART) at equivalent time points, analyzing CSF cellular counts as well as a variety of cytokines, chemokines, and macrophage activation markers.

## METHODS

### Sample Acquisition

**Figure 1.**
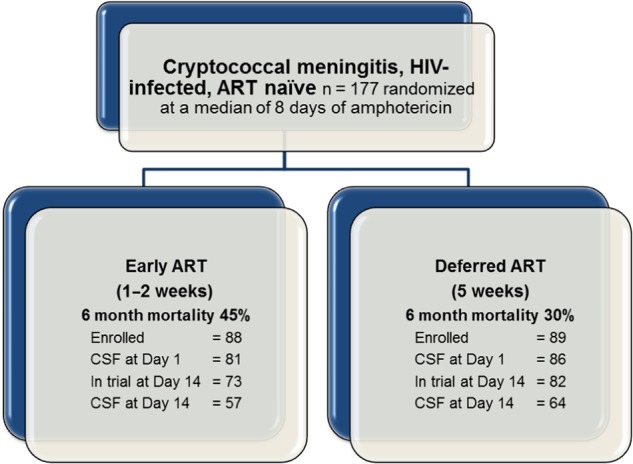
Summary of participants and cerebrospinal fluid (CSF) sampling in the Cryptococcal Optimal ART Timing (COAT) trial. Abbreviations: ART, antiretroviral therapy; HIV, human immunodeficiency virus.

Participants were enrolled in the COAT trial between November 2010 and April 2012 at 3 sites: Kampala, Uganda; Mbarara, Uganda; and Cape Town, South Africa (clinicaltrials.gov NCT01075152); the details of which are reported elsewhere [10]. Briefly, 177 ART-naive persons with cryptococcal meningitis were randomized to receive early or deferred ART initiation (7–13 days or 5 ± 1 weeks postcryptococcal meningitis diagnosis, respectively) (Figure 1). All participants provided written informed consent, and site-specific institutional review board approvals were obtained. As per trial protocol, a lumbar puncture (LP) was performed at diagnosis (amphotericin day 1), trial randomization (median amphotericin day 8, interquartile range [IQR], 7–8), completion of amphotericin therapy (amphotericin day 14, median 6 days ART [IQR, 6–7] in the early ART arm), and additionally at clinicians' discretion, to control elevated intracranial pressure. Serum samples were taken at the same time points, and additionally 21 days after diagnosis. Excess serum and CSF specimens were centrifuged, and supernatant frozen at −80°C.

### Laboratory Procedures

CSF cells and chemistry analysis were measured on site in laboratories participating in external quality assurance testing. Cryopreserved CSF and serum samples were shipped on dry ice (−20°C), thawed, and analyzed in batches at the University of Minnesota. Luminex magnetic bead technology (Bio-Rad Laboratories, Hercules, California) was used to analyze 19 cytokines and chemokines (granulocyte colony-stimulating factor [G-CSF], granulocyte macrophage colony-stimulating factor [GM-CSF], interferon-γ, tumor necrosis factor [TNF]–α, interleukin [IL]–1β, IL-2, IL-4, IL-5, IL-6, IL-7, IL-8, IL-10, IL-12, IL-13, IL-17, MCP-1 [CCL2], macrophage inflammatory protein [MIP]–1α [CCL3], MIP-1β [CCL4], VEGF) according to manufacturers' protocols. Three soluble markers of macrophage activation (sCD163, sCD14, CCL22) were also measured using commercial enzyme-linked immunosorbent assay kits (R&D Systems, Minneapolis, Minnesota). C-reactive protein (CRP) was run at the University of Minnesota Medical Center clinical laboratory.

### Analysis

Demographics and other baseline characteristics are summarized with proportions and median values presented with IQRs. Biomarkers analyzed as continuous variables were log_2_ transformed for normalization, analyzed, and then back-transformed to geometric mean values. All “mean” biomarkers values are geometric means. Low (“out of range”) measurements were set to half of the manufacturer's listed assay limit of detection (LOD). CSF biomarkers with substantial proportions (≥40%) of undetectable values at diagnosis (IL-2, IL-1β, IL-5, CCL22) were analyzed as categorical variables: “detectable” (values greater than the LOD) versus “nondetectable (values lower than the LOD). CSF WCC was analyzed in terms of absolute values, and also classified as “normal” versus “elevated” (<5 vs ≥5 cells/µL, respectively). Means and proportions were compared between trial arms (early vs deferred ART) at equivalent time points. Biomarkers were also compared between participants with CSF WCC <5 cells/µL and those with CSF WCC ≥5 cells/µL at amphotericin day 8 (COAT randomization), as differential outcomes occurred among this group in the COAT trial [10]. Randomization for the COAT trial was stratified by site. However, due to significant differences between sites in terms of baseline characteristics (specifically higher CD4 and CSF white cells in Cape Town), additional subgroup analyses were performed for participants enrolled in Kampala—the largest site where the majority of mortality occurred. Overall tests of heterogeneity (interaction tests) evaluating biomarker treatment differences across participants in Kampala versus the other 2 sites are provided. Analyses were performed using SAS 9.3 (SAS Institute, Cary, North Carolina) with χ^2^ tests, Fisher's exact, and linear regression models as appropriate; plots were generated with R statistical software 2.10.1 (www.R-project.org).

## RESULTS

### Number of Participants and Samples Available

Of the 177 participants enrolled in the COAT trial, 155 were alive and remained enrolled at amphotericin day 14 (Figure 1). CSF WCCs were measured for 166, 146, and 120 participants at amphotericin days 1, 8, and 14, respectively. Participants missing CSF WCC measurements typically had blood-stained CSF. Stored CSF specimens were available for 167, 115, and 121 participants, and stored serum samples were available for 168, 161, and 151 at the same time points; a further 136 participants had serum available 21 days after diagnosis. Due to the low volume of stored CSF at the Mbarara site, it was not possible to measure concentrations of the 3 macrophage-specific biomarkers (sCD163, sCD14, CCL22) in this group of participants.

### Participant Characteristics

Details of the clinical cohort have been previously reported [10]. Median CD4 count for COAT participants with samples available was 28 (IQR, 11–75) cells/µL and median HIV viral load 5.5 (IQR, 5.2–5.8) log_10_ copies/mL. There were no significant differences between study arms in baseline demographics and markers of CM severity (eg, Glasgow Coma Scale score or fungal burden) (Table 1). However, there were clear differences in participants between study sites. Participants in Kampala had significantly lower CD4 cell counts at enrollment and were more likely to have CSF WCC <5 cells/µL throughout the 14-day induction antifungal therapy period (Table 2). Through 14 days, mortality rates were 17%, 6% and 0%, for Kampala, Mbarara, and Cape Town, respectively, among patients surviving to trial enrollment and randomization (median day 8).

**Table 1.** Baseline Characteristics by Cryptococcal Optimal ART Timing (COAT) Trial Arm Among Individuals With CSF Biomarkers Measured at Cryptococcal Meningitis Diagnosis

Characteristic	Early ART (n = 81)	Deferred ART (n = 86)
Age, years	35 (28, 40)	36 (30, 40)
Male gender	40 (49%)	45 (52%)
Weight, kg	53.0 (46.5, 59.0)	54.1 (48.0, 60.7)
Glasgow Comal scale <15	21 (26%)	26 (30%)
CSF opening pressure, mmH_2_O	285 (190, 360)	260 (180, 418)
CSF quantitative culture, log_10_ CFU/mL	5.3 (4.3, 5.7)	4.8 (3.8, 5.4)
CSF cryptococcal antigen titer	8000 (2000, 16 000)	4000 (1000, 16 000)
CSF white cells, cells/µL	10 (≤4, 85)	29 (≤4, 110)
CSF protein, mg/dL	110 (50, 188)	111 (64, 182)
On tuberculosis treatment	21 (26%)	18 (21%)
CD4 count, cells/µL	28 (10, 70)	29 (11, 76)
HIV RNA, log_10_ copies/mL	5.5 (5.2, 5.8)	5.5 (5.3, 5.8)

Data are median (P25, P75) or N (%). The early antiretroviral therapy (ART) group initiated ART at a median of 8 (interquartile range [IQR], 7–8) days from cryptococcal diagnosis; the deferred ART group initiated ART at a median of 36 (IQR, 34–39) days from diagnosis. No significant differences were noted at baseline between trial arms.

Abbreviations: CFU, colony-forming units; CSF, cerebrospinal fluid; HIV, human immunodeficiency virus.

**Table 2.** Differences in Baseline Characteristics and Outcomes Between Study Sites in the COAT Trial

Characteristic or Outcome	Kampala N = 115	Mbarara N = 35	Cape Town N = 27	*P* Value
At cryptococcal meningitis diagnosis				
CD4 cells/µL	17 (7, 69)	36 (14, 74)	67 (43, 88)	<.001
HIV viral load, log_10_ copies/mL	5.5 (5.3, 5.7)	5.5 (5.2, 5.9)	5.5 (5.1, 5.9)	.68
Glasgow Coma Scale score <15	34 (30%)	10 (29%)	3 (11%)	.14
Quantitative CSF culture, log_10_ CFU/mL	5.0 (3.9, 5.5)	5.2 (4.3, 5.6)	4.8 (2.8, 6.0)	.58
CSF white cells, day 1	30 (≤4, 108)	5 (0, 10)	82 (17, 313)	<.001
CSF white cells ≥5 cells/µL				
At diagnosis (amphotericin day 1)	61 (56.5%)	20 (62.5%)	22 (85%)	.03
At randomization (amphotericin day 8)	47 (49%)	9 (53%)	23 (92%)	<.001
At end of induction therapy (day 14)	24 (33%)	11 (50%)	23 (92%)	<.001
Sterile CSF at end of induction therapy	39 (48%)	13 (59%)	16 (67%)	.24
Mortality				
Within 14 d of diagnosis	20 (17%)	2 (5.7%)	0 (0.0%)	.02
Within 30 d of diagnosis	32 (28%)	7 (20%)	1 (3.7%)	.02
Within 46 wk of diagnosis	49 (43%)	13 (37%)	8 (30%)	.44

Data are median (P25, P75) or N (%). *P* values are from Kruskal–Wallace or χ^2^ tests as appropriate.

Abbreviations: CFU, colony-forming unit; COAT, Cryptococcal Optimal ART Timing; CSF, cerebrospinal fluid; HIV, human immunodeficiency virus.

### Effect of Early ART on CSF Cellular Infiltrate

**Figure 2.**
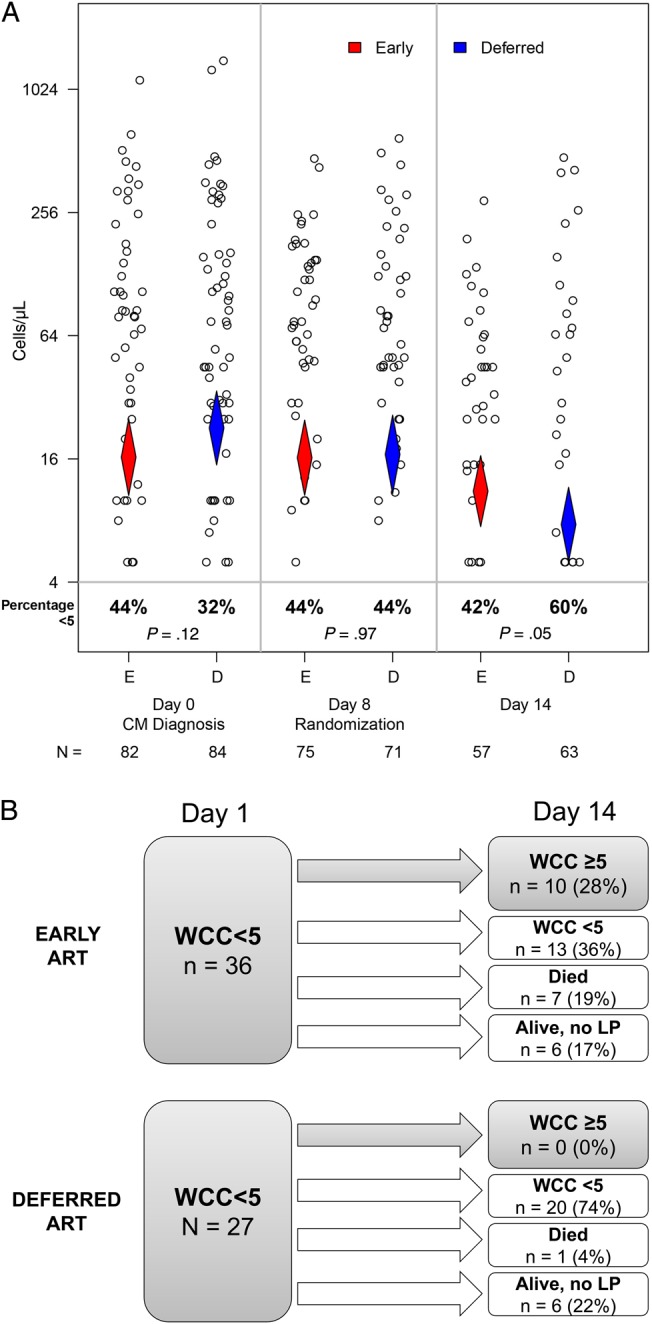
*A*, Differences in cerebrospinal fluid (CSF) white cell count (WCC) between trial arms during the 14 days of amphotericin therapy. WCCs are shown as dots, diamonds indicate the geometric mean and 95% confidence interval (CI), the percentage of participants with WCC <5 are shown below along with total numbers. There were no significant differences in geometric mean CSF WCC between early antiretroviral therapy (ART) and deferred ART. However, at the end of antifungal induction therapy (amphotericin day 14), the percentage of participants with CSF WCC <5 cells/µL was significantly lower in the early ART (42%) compared with the deferred ART group (60%; χ^2^ test, *P* = .047). *B*, Effect of early ART initiation on CSF WCC after antifungal induction therapy in participants with CSF WCC <5/µL at cryptococcal meningitis (CM) diagnosis. A similar number of participants in each trial arm had CSF WCC <5/µL at presentation (left). However, a significantly increased proportion of participants in the early ART group had WCC ≥5/µL at day 14 (top right) compared to deferred ART (bottom right) (Fisher's exact *P* = .0008).

At presentation (amphotericin day 1) and at COAT randomization (amphotericin day 8), there were no differences between study arms in median CSF WCC, or the proportion of participants with a raised CSF WCC (≥5 cells/µL). However, by amphotericin day 14, there were significantly more participants in the early ART group with raised CSF WCC compared to the deferred group (58% vs 40%; *P* = .047; Figure 2*A*). At 14 days of amphotericin, participants in the early ART group had received ART for a median of 6 days (IQR, 6–7). Amphotericin day-14 differences in CSF WCC were predominantly driven by increasing WCCs in those with CSF WCC <5/µL at day 1 who received early ART. Raised CSF WCC at amphotericin day 14 occurred in 28% (10/36) of persons with CSF WCC <5/µL at day 1 in the early ART arm, but in 0% (0/27) with CSF WCC <5/µL at day 1 in the deferred ART arm (*P* = .002) (Figure 2*B*).

### Effect of Early ART on CSF Soluble Inflammatory Markers

**Figure 3.**
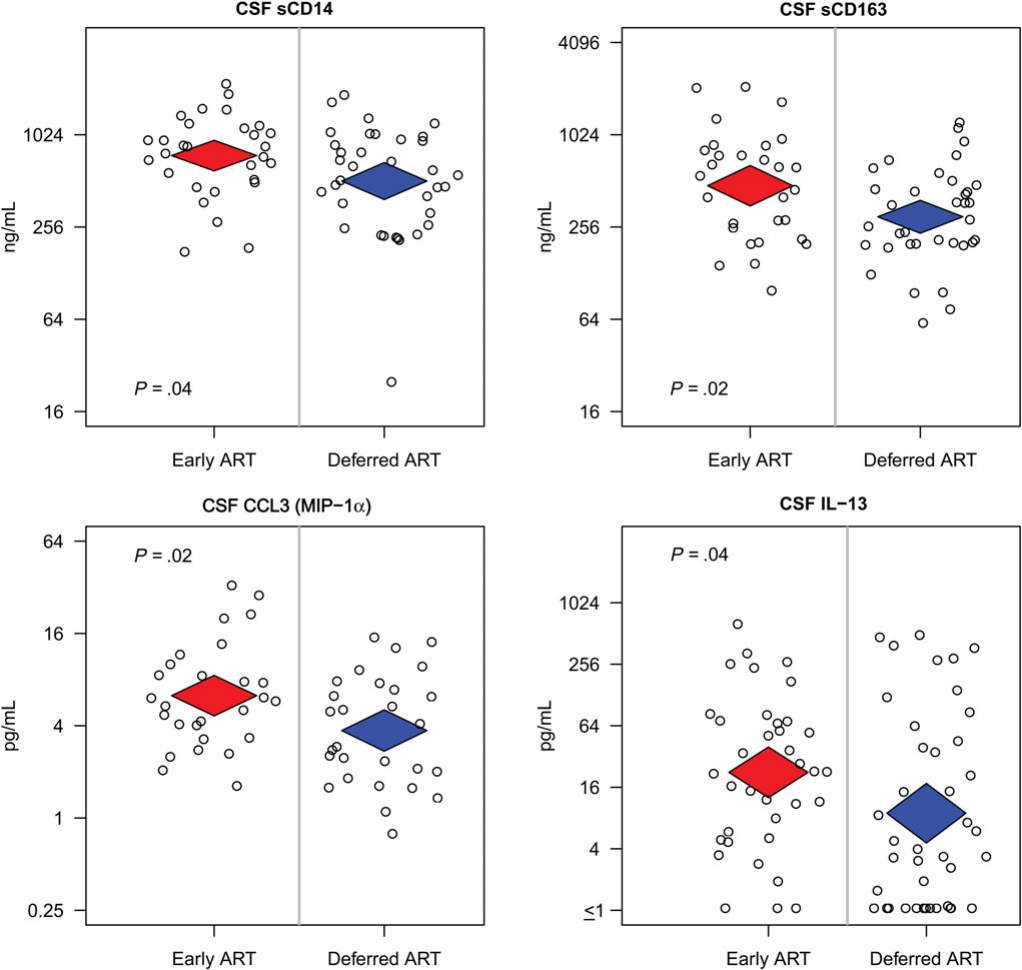
Differences in day 14 cerebrospinal fluid (CSF) biomarkers between Cryptococcal Optimal ART Timing (COAT) trial groups enrolled in Kampala, Uganda. Significantly increased concentration of interleukin 13 (IL-13), macrophage inflammatory protein (MIP)-1α/CCL3, and the macrophage activation markers sCD14 and sCD163, were noted at day 14 in participants in the early antiretroviral therapy (ART) group compared to deferred ART group in Kampala. *P* values from linear regression models. CSF IL-13 was undetectable in 8% (3/36) in the early ART and 29% (12/41) in the deferred ART (Fisher's exact *P* = .02.).

CSF levels of 19 different cytokines/chemokines and 3 soluble markers of macrophage activation were analyzed over the induction period. Overall, during the 14-day induction antifungal therapy, there was a >50% reduction in multiple CSF cytokines/chemokines, including interferon-γ, IL-6, IL-8, IL-10, and TNF-α (*P* < .01 for each). No differences in the concentration of CSF biomarkers were observed between treatment groups in the overall cohort (Supplementary Table 1*A*), although when testing for heterogeneity of the immune response to early ART (via interaction *P* values), participants from Kampala (the largest site) had multiple potential differences in the response to early ART versus the other sites. A subgroup analysis of the randomized Kampala participants with available CSF at amphotericin day 14 (n = 77) showed increased CSF concentration of the macrophage markers sCD14 and sCD163 in the early ART group (sCD14 mean 749 vs 511 ng/mL, *P* = .04; sCD163 mean 476 vs 299 ng/mL, *P* = .02; early vs deferred ART, respectively) as well as the chemokine MIP-1α (MIP-1α mean 6.3 vs 3.7 pg/mL; *P* = .02) (Figure 3, Supplementary Table 1*B*). In addition, Kampala participants receiving early ART also had increased day-14 CSF concentrations of the T helper 2 (Th2) cytokine IL-13 (IL-13 mean 22.4 vs 8.9 pg/mL; *P* = .04). IL-13 concentrations in the early ART arm in Kampala participants at day 14 were >2 fold higher than the concentrations at diagnosis, and were driven predominantly by a greater proportion with detectable CSF IL-13 with early ART (92% vs 71% detectable, early, and deferred ART, respectively; *P* = .02). Additionally, at this same time point, participants receiving early ART were significantly less likely to have detectable IL-2 in their CSF (>1.1 pg/mL) compared with deferred ART (50% vs 76%, respectively; *P* = .02).

### Effect of ART on Serum Soluble Inflammatory Markers

No significant differences were observed in the concentration of serum cytokines and chemokines between trial arms in the first 14 days (Supplementary Table 2). However, at day 14, individuals in the early ART arm had significantly lower serum CRP compared with the deferred ART arm (mean 89 (95% confidence interval [CI], 73–109) versus 123 (95% CI, 105–142) mg/L, *P* = .01; early vs deferred ART, respectively). In contrast, at day 21, CRP in the early arm was significantly higher than in the deferred arm (mean 51 (95% CI, 39–67) versus 33 (95% CI, 25–45) mg/L, *P* = .04; early vs deferred ART, respectively; Supplementary Table 3). This resulted in 2 distinct patterns of CRP change during the first 3 weeks of the trial. With deferred ART, CRP gradually increased throughout amphotericin treatment, peaked at day 14, and fell rapidly afterward. Conversely, in the early ART group, CRP also rose during the first week of amphotericin therapy but appeared to plateau after ART was initiated at day 8, returning more slowly to baseline values (Supplementary Figure 1). In addition, the early ART group had lower serum sCD163 concentration at day 21 (mean 799 vs 1009 ng/mL, *P* = .01; early vs deferred ART, respectively).

### Associations With low CSF WCC

**Figure 4.**
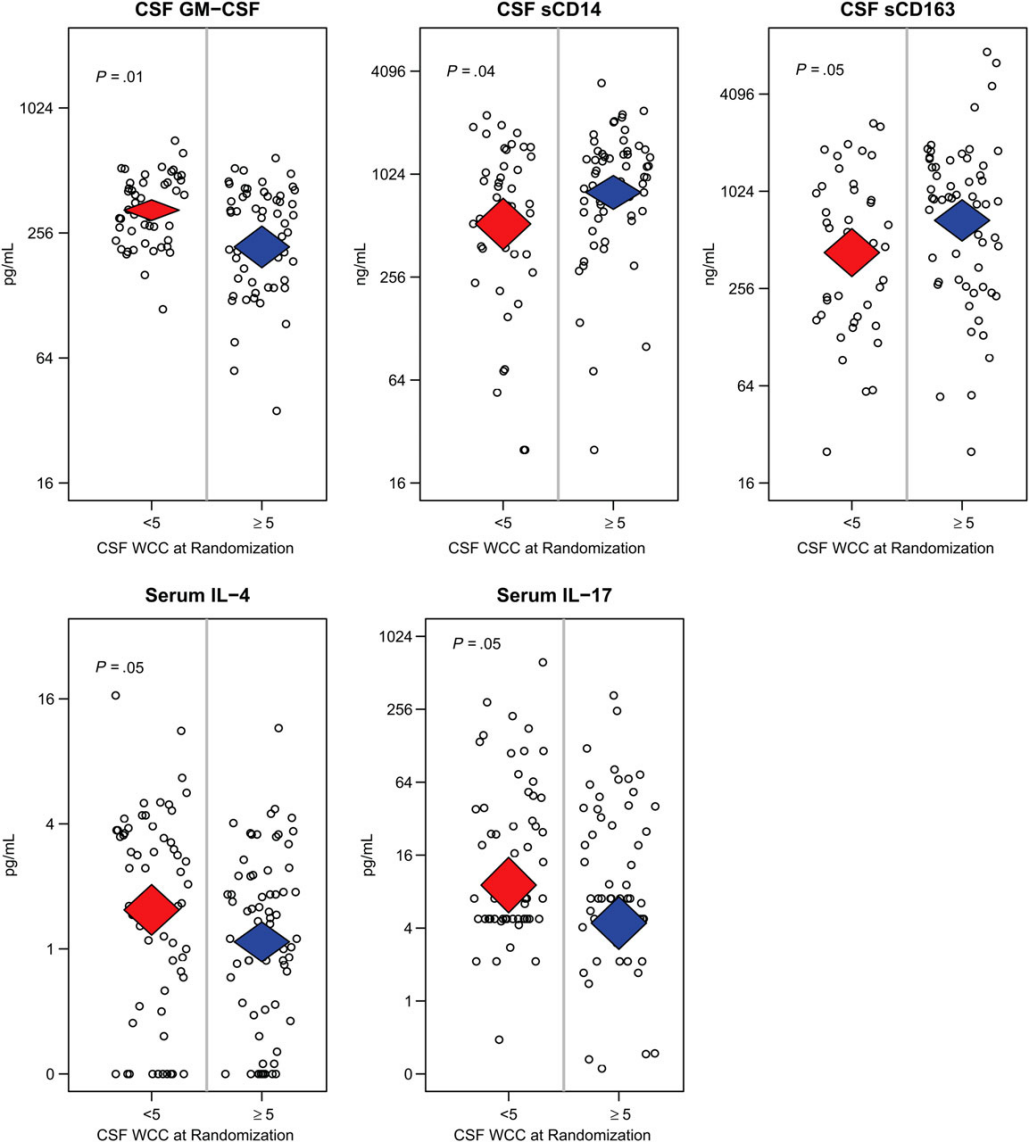
Differences in serum and cerebrospinal fluid (CSF) biomarkers between subjects with normal or raised CSF white cell count (WCC) at Cryptococcal Optimal ART Timing randomization (median amphotericin day 8). Participants who had a normal CSF WCC (<5/µL) at randomization had significantly higher serum interleukin (IL)-4, IL-17, and CSF granulocyte-macrophage-colony-stimulating factor (GM-CSF) levels, but significantly lower CSF sCD14 and sCD163 levels, compared to participants with raised CSF WCC (≥5/μL). Supplementary Table 4*A* and 4*B* present detailed data for CSF and blood, respectively.

In the COAT trial, the difference in survival between early and deferred ART was particularly marked in individuals who had a CSF WCC <5/µL at randomization (amphotericin day 8) [10]. To further characterize these participants' immune phenotype, we compared CSF and serum biomarker concentrations between participants with normal (<5/µL) and elevated (≥5/µL) CSF WCC at this time point. Participants with WCC <5/µL at randomization were significantly less likely to have detectable CCL22 in their CSF compared to those with WCC ≥5/µL (63% vs 81%, respectively; *P* = .04), they also had significantly increased CSF GM-CSF (*P* < .01) and significantly lower CSF concentrations of the macrophage activation markers sCD14 (*P* = .05) and sCD163 (*P* = .04). Participants with CSF WCC <5/µL at randomization also had increased serum concentrations of the Th2 cytokine IL-4 (*P* = .05), and the Th17 cytokine IL-17 (*P* = .05) (Figure 4). Among those randomized to early ART, day-14 CSF biomarkers did not statistically differ between participants with normal or raised CSF WCC at randomization.

## DISCUSSION

The COAT trial demonstrated that in patients with cryptococcal meningitis, initiation of ART within the first 1–2 weeks of meningitis treatment resulted in 15% increased mortality compared with deferred ART initiation at 4–6 weeks [10]. The excess deaths associated with early ART in the trial occurred primarily within the first 2–5 weeks after meningitis diagnosis and were particularly increased among participants with a paucicellular CSF response at randomization (ie, WCC <5/µL, 32% higher mortality with early ART) [10]. This study examines the effect of early ART initiation on the host immune response in an attempt to explain the pathophysiology behind the excess mortality observed in the COAT trial.

Early ART initiation was associated with an increased proportion of participants with CSF white cell infiltrate by day 14 of amphotericin. This was particularly marked among participants who had an initial paucity of CSF white cells (<5/µL). By day 14, 28% of such participants in the early ART arm (with CSF white cells<5/µL) had developed a CSF cellular infiltrate and an additional 19% had died, compared with 0% with cellular infiltrate in the deferred ART arm and only 4% mortality. We hypothesize that this influx of cells into the CSF represents a detrimental, ART-driven, immune response that may explain the excess mortality observed with early ART initiation in CM. Furthermore, in Kampala, persons receiving early ART had increased CSF concentrations of sCD163, sCD14, and MIP-1α at day 14. This is consistent with increased macrophage activation in the central nervous system (CNS), and the increased CSF IL-13 suggests a Th2 skew to the immune response in those receiving early ART [13, 14].

Th2 immune responses have been shown to be detrimental during infection with intracellular organisms such as *Cryptococcus neoformans*, whereas Th1 responses are protective [13, 14]. In the overall cohort, mean CSF IL-13 concentrations increased from diagnosis to day 14 by approximately 20% with early ART, and by approximately 100% with early ART in Kampala participants. Among persons deferring ART, CSF IL-13 levels declined by approximately 40% over 14 days. Conversely, CSF concentrations of interferon-γ gradually declined by approximately 50% in both arms over 14 days as the infection was treated. Given that the majority of mortality occurred in Kampala, the finding that early ART was associated with increased CNS macrophage activation and Th2 response (just 6–7 days after ART initiation) in Kampala participants further supports the hypothesis that ART-associated immunopathology was likely responsible for the increased mortality observed in this trial.

In the COAT trial, there was no statistically significant increase in paradoxical CM–IRIS with early ART initiation (20%) versus deferred ART (13%) (*P* = .32) [10]. This is in contrast to the increased incidence of tuberculosis-IRIS reported with early ART in HIV-associated tuberculosis [15–17]. However, the case definition of paradoxical CM-IRIS specifies that persons must undergo a pattern of clinical improvement prior to their ART-associated clinical deterioration [18]. In the COAT trial, early ART was often initiated prior to the resolution of meningitis symptoms, making it difficult to identify such a biphasic clinical course with distinct second events. Yet, the hypothesis that early ART resulted in adverse inflammatory responses is supported by the ART-associated CSF cellular infiltrate occurring exclusively among participants who failed to mount a CSF leukocyte pleocytosis initially (WCC <5/µL). This was the same subgroup of participants found to be at high risk of mortality with early ART in the COAT trial, and the same group previously shown to be at >3-fold increased risk of CM-IRIS [10–12]. Further examination of participants in this study with a paucicellular CSF response prior to ART initiation showed they also had significantly reduced CSF macrophage activation markers (sCD14, sCD163) and significantly increased serum IL-4 and IL-17 concentrations prior to ART. Pre-ART elevations of IL-4 and IL-17 have previously been reported in persons who subsequently developed CM-IRIS after starting ART [19]. It is possible this group in the COAT trial may have responded similarly.

In the Kampala subgroup, we detected increased CSF markers of macrophage activation (sCD14 and sCD163) among participants receiving early ART. CD163 is a haptoglobin-hemoglobin scavenging receptor found on the surface of monocytes, macrophages, and activated microglia [20]; it is cleaved from the cell surface in response to cellular activation, and concentrations of soluble CD163 (sCD163) are considered an accurate reflection of macrophage and monocyte activation [21–23]. In HIV infection, serum concentrations are positively correlated with HIV-1 viral load, inversely correlated with CD4 count, and fall following ART initiation [24]. In this study, sCD163 levels were also significantly reduced in serum among individuals receiving early ART but were significantly increased in CSF. This suggests an early differential ART effect on the host immune response at the site of disease. Monocyte or macrophages have previously been implicated in the pathophysiology of paradoxical tuberculosis-IRIS [25–28]. The increased CSF sCD163 levels noted herein imply that CNS macrophage and/or microglia activation may play a role in the early ART-associated mortality observed.

CD14 is also found on monocytes, macrophages, and activated microglia, and functions as a receptor for lipopolysaccharide as well as glucuronoxylomannan cryptococcal antigen [29–31]. Similar to sCD163, CD14 can also be released from the cell surface in response to a range of stimuli, and thereafter is detectable as soluble CD14 (sCD14) [29]. Serum concentrations of sCD14 are elevated in HIV-1 infection, possibly due to translocation of microbial components through the intestinal wall, and sCD14 levels are associated with increased mortality [32]. Raised CSF sCD14 has also been reported in bacterial meningitis, both in hospitalized patients and mouse models [33, 34]. In these studies, CSF sCD14 elevations appeared to result from increased intrathecal production by infiltrating blood white cells rather than diffusion across the blood-brain barrier [33, 34]. In addition to being a marker of monocyte-macrophage activation, sCD14 augments the CSF immune response, leading to increased proinflammatory cytokines IL-6 and TNF-α [34]. Elevated CSF MIP-1α/CCL3 also occurs in bacterial and tuberculosis meningitis [35, 36], and an increased CSF CCL3/CXCL10 ratio prior to ART initiation is associated with future cryptococcal-IRIS [37]. In addition to its chemotactic activities, MIP-1α also modulates the immune response by stimulating proinflammatory cytokine production from a variety of cells [38]. It is possible that sCD14 and MIP-1α/CCL3 may play a similar role here, not only reflecting increased macrophage activation within the CNS, but providing insight into the mechanism by which ART initiation may lead to a detrimental proinflammatory immune response.

Previous cryptococcal-IRIS studies have demonstrated CSF elevations in many proinflammatory cytokines (eg, TNF-α, interferon-γ, G-CSF, IL-6) [12]. In this trial, IL-13 was the only T-cell–related cytokine increased with early ART. However, CSF was only systematically sampled 6 days after ART initiation. Had later samples been collected, it is possible that an evolution in CNS immune response may have been demonstrated: from cellular infiltrate, increased macrophage activation and chemokine secretion, to widespread elevation of proinflammatory cytokines. In a separate COAT trial analysis using flow cytometry, highly activated CD8^+^ T cells predominated in CSF at diagnosis (median CD4:CD8 ratio = 0.06), yet at paradoxical-IRIS, CD4^+^ T cells had increased in CSF (median CD4:CD8 ratio = 0.33) [39].

In contrast to the CSF effects observed with early ART, there was no evidence to suggest early ART led to increased systemic inflammatory response. Serum cytokine and chemokine concentrations were not elevated, and CRP initially decreased. This may reflect compartmentalization of immune response, which has been reported in tuberculosis meningitis paradoxical-IRIS, where individuals had marked elevation in CSF proinflammatory cytokines but little change in serum [40]. However, in our study at 21 days, serum CRP was higher among persons receiving early ART (median 14 days of ART), possibly reflecting the evolution of inflammatory response on the path to paradoxical cryptococcal-IRIS in certain patients [19, 41].

There are limitations of this study. Due to the COAT trial being halted prematurely, these immunologic analyses are underpowered. Reported associations are significant in terms of calculated *P* values, but due to the exploratory nature of the study, no adjustment was made for multiple comparisons. In addition, some of the significant findings reported here are from subgroup analyses; this was considered appropriate given the heterogeneity in demographics, clinical parameters, and immune response between the 3 trial sites. Finally, CSF was not available for all study subjects at day 14 (due to early mortality, approximately 10% LP refusal, and approximately 8% normalized intracranial pressure with a small volume of CSF collected); however, CSF was available for over 80% of trial participants.

Despite these limitations, this study provides a unique insight into the immunologic changes that occurred when patients with CM received early ART initiation. Given the findings of the COAT trial, it is unlikely a similar trial should or will be repeated. The data presented herein provide evidence suggesting early ART led to a CSF cellular infiltrate, CNS macrophage/microglial activation, and skew toward a Th2 immune response. These findings support the hypothesis that the increased mortality associated with early ART in the COAT trial was immunologically mediated with compartmentalized neuroinflammation.

## Supplementary Data

Supplementary materials are available at *The Journal of Infectious Diseases* online (http://jid.oxfordjournals.org). Supplementary materials consist of data provided by the author that are published to benefit the reader. The posted materials are not copyedited. The contents of all supplementary data are the sole responsibility of the authors. Questions or messages regarding errors should be addressed to the author.

Supplementary Data
