# Supplementary material for: Early ART After Cryptococcal Meningitis Is Associated With Cerebrospinal Fluid Pleocytosis and Macrophage Activation in a Multisite Randomized Trial
Source: J Infect Dis. 2015 Feb 4;212(5):769–78. doi: 10.1093/infdis/jiv067 (PMC4527410; doi:10.1093/infdis/jiv067)
Supplement: Supplementary Data [file supp_212_5_769__index.html]

Early ART after Cryptococcal Meningitis is associated with Cerebrospinal Fluid Pleocytosis and Macrophage Activation in a Multisite Randomized Trial — Early ART After Cryptococcal Meningitis Is Associated With Cerebrospinal Fluid Pleocytosis and Macrophage Activation in a Multisite Randomized Trial — Early ART After Cryptococcal Meningitis Is Associated With Cerebrospinal Fluid Pleocytosis and Macrophage Activation in a Multisite Randomized Trial — Supplementary Data 

# Early ART After Cryptococcal Meningitis Is Associated With Cerebrospinal Fluid Pleocytosis and Macrophage Activation in a Multisite Randomized Trial

## Supplementary Data

Supplementary Data

**Files in this Data Supplement:**

- Supplementary Data - Pdf file
