# Supplementary material for: Early ART After Cryptococcal Meningitis Is Associated With Cerebrospinal Fluid Pleocytosis and Macrophage Activation in a Multisite Randomized Trial
Source: J Infect Dis. 2015 Feb 4;212(5):769–78. doi: 10.1093/infdis/jiv067 (PMC4527410; doi:10.1093/infdis/jiv067)
Supplement: Supplementary Data [file supp_jiv067_jiv067supp.pdf]

Supplemental Figure 1: Differences in serum CRP between trial arms over the first 21 days of antifungal treatment. Participants' CRP values are shown as dots. Diamonds represent geometric mean and 95% CI. Numbers of participants per group are shown below. CRP levels increased during the 14 days of amphotericin therapy and declined afterwards. In the group that received early ART initiation, CRP was significantly lower at day 14 (P=0.01) but significantly higher at day 21 (P=.04) than with deferred ART.

Supplementary Table 1a – Differences in CSF biomarkers between trial arms at day 14 (whole cohort). No significant differences are noted between early and deferred ART. Biomarker values are geometric mean values from log2 transformation. P-values are from linear regression or Chi-square tests.

CSF Biomarker	Interaction P-value*	Early ART		Deferred ART		P-value
		N	Mean (95% CI)	N	Mean (95% CI)	
White cell count, cells/μL	0.82	57	14.0 (9.8 - 20.0)	63	9.9 (6.6 - 14.7)	0.20
G-CSF, pg/mL	0.67	57	59.0 (44.4 - 78.4)	64	61.2 (45.6 - 82.1)	0.86
GM-CSF, pg/mL	0.31	57	256 (195 - 337)	64	253 (190 - 337)	0.95
IFN-γ, pg-mL	0.02	57	19.3 (14.1 - 26.5)	64	20.7 (15.2 - 28.2)	0.75
TNF-α, pg/mL	0.09	57	5.3 (4.4 - 6.5)	64	5.5 (4.4 - 6.9)	0.79
VEGF, pg/mL	0.82	57	57.5 (38.0 - 86.9)	64	57.4 (39.4 - 83.5)	0.99
IL-4, pg/mL	<.01	57	0.5 (0.4 - 0.5)	64	0.5 (0.4 - 0.6)	0.43
IL-6, pg/mL	0.05	57	41.7 (28.3 - 61.5)	64	35.5 (21.5 - 58.4)	0.62
IL-7, pg/mL	0.56	57	6.3 (4.5 - 8.8)	64	5.4 (3.8 - 7.7)	0.55
IL-8, pg/mL	0.30	57	228 (171 - 305)	64	211 (160 - 279)	0.70
IL-10, pg/mL	0.24	57	6.1 (5.2 - 7.2)	64	5.5 (4.3 - 7.0)	0.50
IL-12, pg/mL	0.15	57	9.8 (7.8 - 12.3)	64	8.9 (6.9 - 11.6)	0.61
IL-13, pg/mL	0.15	57	24.7 (15.3 - 40.0)	64	14.5 (8.5 - 24.7)	0.15
IL-17, pg/mL	0.32	57	4.6 (3.0 - 7.1)	64	4.1 (2.7 - 6.4)	0.71
CCL2 / MCP-1, pg/mL	0.45	57	408 (288 - 578)	64	420 (295 - 599)	0.90
CCL3 / MIP-1α, pg/mL	0.01	48	6.1 (4.6 - 8.1)	51	5.6 (4.3 - 7.3)	0.66
CCL4 / MIP-1b, pg/mL	0.12	57	92.8 (77.4 - 111)	64	86.2 (70.5 - 106)	0.60
sCD14, ng/mL	0.02	39	706 (586 - 849)	48	597 (479 - 745)	0.27
sCD163, ng/mL	<0.001	39	479 (369 - 623)	48	428 (332 - 551)	0.55
			N (%) Detectable		N (%) Detectable	
IL-1β, pg/mL	0.01	57	17 (30%)	64	17 (27%)	0.69
IL-2, pg/mL	0.08	57	33 (58%)	64	46 (72%)	0.11
IL-5, pg/mL	0.35	57	23 (40%)	64	30 (47%)	0.47
CCL22 / MDC, pg/mL	0.77	39	26 (67%)	48	31 (65%)	0.84

*P-value for the interaction between the Kampala site and ART group. A significant p-value implies there may have been a differential response to early vs. deferred ART by site. sCD14 and sCD163 were not measured on Mbarara specimens, due to limited of volume, so for these the interaction is a comparison of Kampala vs. Cape Town sites.

G-CSF = granulocyte colony stimulating factor; GM-CSF = granulocyte-monocyte colony stimulating factor; IFN = interferon; TNF = tumor necrosis factor; VEGF = vascular endothelial growth factor; IL = interleukin; CCL2 = MCP-1 = monocyte chemoattractant protein-1; CCL3 = MIP1-α = Macrophage inflammatory protein-1 alpha; CCL4 = MIP-1β = Macrophage inflammatory protein-1-beta. CCL22 = MDC = macrophage-derived chemokine

Supplementary Table 1b – Differences in CSF biomarkers between trial arms at day 14 (Kampala). As shown in figure 3, mean IL-13, MIP-1-α/CCL3, sCD14 and sCD163 were significantly higher in the early ART group at day 14 compared to deferred ART among subjects enrolled in Kampala. No significant differences were noted in the other cytokines and chemokines measured. Biomarker values are geometric mean values from log2 transformation. P-values are from linear regression or Chi-square tests.

CSF Biomarker	Early ART		Deferred ART		P-value
	N	Mean (95% CI)	N	Mean (95% CI)	
White cell count, cells/μL	35	12.3 (8.3 - 18.2)	38	8.2 (5.8 - 11.4)	0.12
G-CSF, pg/mL	36	51.7 (37.7 - 71.0)	41	50.3 (34.0 - 74.5)	0.92
GM-CSF, pg/mL	36	252 (201 - 315)	41	290 (256 - 330)	0.27
IFN-γ, pg-mL	36	18.3 (13.1 - 25.6)	41	13.4 (9.5 - 18.7)	0.20
TNF-α, pg/mL	36	4.2 (3.3 - 5.3)	41	3.7 (2.9 - 4.7)	0.48
VEGF, pg/mL	36	66.1 (41.9 - 104.3)	41	68.9 (50.6 - 93.8)	0.88
IL-4, pg/mL	36	0.5 (0.4 - 0.5)	41	0.4 (0.3 - 0.5)	0.28
IL-6, pg/mL	36	38.9 (23.4 - 64.7)	41	20.7 (11.8 - 36.4)	0.11
IL-7, pg/mL	36	4.5 (3.3 - 6.2)	41	3.5 (2.4 - 5.3)	0.37
IL-8, pg/mL	36	207 (142 - 302)	41	164 (121.9 - 222)	0.34
IL-10, pg/mL	36	5.5 (4.5 - 6.7)	41	4.3 (3.3 - 5.7)	0.20
IL-12, pg/mL	36	10.6 (8.5 - 13.3)	41	8.0 (5.9 - 10.8)	0.15
IL-13, pg/mL	36	22.4 (12.8 - 39.3)	41	8.9 (4.6 - 17.3)	0.04
IL-17, pg/mL	36	5.4 (3.4 - 8.5)	41	3.8 (2.2 - 6.4)	0.34
CCL2 / MCP-1, pg/mL	36	382 (246 - 594)	41	342 (217 - 539)	0.74
CCL3 / MIP-1α, pg/mL	27	6.3 (4.7 - 8.5)	28	3.7 (2.7 - 5.0)	0.02
CCL4 / MIP-1b, pg/mL	36	94.3 (73.7 - 121)	41	74.5 (59.1 - 93.9)	0.18
sCD14, ng/mL	29	748.5 (597 - 938)	35	510.5 (388 - 671)	0.04
sCD163, ng/mL	29	476 (353 - 642)	35	299 (234 - 381)	0.02
		N (%) Detectable		N (%) Detectable	
IL-1β, pg/mL	36	15 (42%)	41	9 (22%)	0.06
IL-2, pg/mL	36	18 (50%)	41	31 (76%)	0.02
IL-5, pg/mL	36	14 (39%)	41	16 (39%)	0.99
CCL22 / MDC, pg/mL	29	17 (59%)	35	19 (54%)	0.73

G-CSF = granulocyte colony stimulating factor; GM-CSF = granulocyte-monocyte colony stimulating factor; IFN = interferon; TNF = tumor necrosis factor; VEGF = vascular endothelial growth factor; IL = interleukin; CCL2 = MCP-1 = monocyte chemoattractant protein-1; CCL3 = MIP1-α = Macrophage inflammatory protein-1 alpha; CCL4 = MIP-1β = Macrophage inflammatory protein-1-beta; CCL22 = MDC = macrophage-derived chemokine

Supplementary Table 2 - Differences in serum biomarkers between trial arms at day 14 (whole cohort, all sites). No significant differences are noted in the concentration of serum cytokines and chemokines between early and deferred ART groups at day 14. However, CRP was significantly lower in the early ART group.

Serum Biomarker	Early ART		Deferred ART		P-value
	N	Mean (95% CI)	N	Mean (95% CI)	
G-CSF, pg/mL	73	13.6 (10.9, 16.8)	78	17.5 (13.6, 22.4)	0.13
GM-CSF, pg/mL	73	2.4 (1.7, 3.3)	78	2.4 (1.7, 3.2)	0.99
IFN-γ, pg/mL	73	41.3 (31.6, 54.1)	78	45.9 (34.0, 62.0)	0.61
TNF-α, pg/mL	73	12.6 (9.0, 17.7)	78	18.2 (12.8, 26.0)	0.15
VEGF, pg/mL	73	245 (169, 353)	78	160 (118, 218)	0.08
IL-1β, pg/mL	73	0.9 (0.8, 1.1)	78	1.1 (0.9, 1.3)	0.27
IL-2, pg/mL	73	0.7 (0.6, 0.8)	78	0.8 (0.7, 1.1)	0.19
IL-4, pg/mL	73	0.8 (0.6, 0.9)	78	0.9 (0.7, 1.1)	0.42
IL-5, pg/mL	73	1.3 (1.1, 1.6)	78	1.5 (1.2, 1.9)	0.29
IL-6, pg/mL	73	36.1 (25.1, 51.9)	78	47.3 (34.9, 64.2)	0.26
IL-7, pg/mL	73	9.8 (8.4, 11.4)	78	9.8 (8.5, 11.3)	0.99
IL-8, pg/mL	73	49.0 (40.3, 59.5)	78	39.4 (31.7, 48.9)	0.15
IL-10, pg/mL	73	3.0 (2.3, 3.9)	78	3.5 (2.5, 5.1)	0.47
IL-12, pg/mL	73	10.9 (9.1, 13.1)	78	13.3 (11.2, 15.8)	0.13
IL-13, pg/mL	73	3.1 (2.5, 3.7)	78	3.2 (2.6, 4.0)	0.69
IL-17, pg/mL	73	5.2 (3.3, 8.3)	78	5.5 (3.4, 8.7)	0.89
CCL2 / MCP-1, pg/mL	73	47.4 (36.3, 61.8)	78	60.5 (46.9, 78.2)	0.20
CCL3 / MIP-1α, pg/mL	73	12.6 (8.4, 19.0)	78	10.9 (7.6, 15.7)	0.60
CCL4 / MIP-1β, pg/mL	73	200 (147, 271)	78	187 (138, 253)	0.76
sCD14, ng/mL	73	4.6 (4.2, 5.1)	78	4.8 (4.4, 5.3)	0.56
sCD163, ng/mL	73	1014 (869, 1184)	78	1141 (984, 1323)	0.28
CRP, mg/L	73	89.0 (72.7, 109)	80	123 (105, 145)	0.01

Biomarker values are geometric mean values from log2 transformation. P-values are from linear regression.

G-CSF = granulocyte colony stimulating factor; GM-CSF = granulocyte-monocyte colony stimulating factor; IFN = interferon; TNF = tumor necrosis factor; VEGF = vascular endothelial growth factor; IL = interleukin; CCL2 = MCP-1 = monocyte chemoattractant protein-1; CCL3 = MIP1-α = Macrophage inflammatory protein-1 alpha; CCL4 = MIP-1β = Macrophage inflammatory protein-1-beta; CRP = C-reactive protein.

Supplementary Table 3 - Differences in serum biomarkers between trial arms at day 21 (whole cohort). No significant differences are noted in the concentration of serum cytokines and chemokines between early and deferred ART groups at day 21. However, CRP was significantly higher in the early ART group and sCD163 was significantly lower.

**Serum Biomarker**	**Early ART**		**Deferred ART**		**P-value**
	N	Mean (95% CI)	N	Mean (95% CI)	
G-CSF, pg/mL	63	14.3 (10.6, 19.3)	73	10.9 (8.2, 14.5)	0.20
GM-CSF, pg/mL	63	1.7 (1.2, 2.4)	73	1.4 (1.0, 1.8)	0.39
IFN-γ, pg/mL	63	21.5 (14.0, 33.0)	73	14.5 (9.8, 21.4)	0.18
TNF-α, pg/mL	63	11.8 (7.8, 17.8)	73	9.2 (6.4, 13.2)	0.37
VEGF, pg/mL	63	152 (93.8, 245)	73	124 (82.1, 188)	0.54
IL-1β, pg/mL	63	1.3 (1.1, 1.5)	73	1.1 (0.9, 1.2)	0.16
IL-2, pg/mL	63	0.7 (0.6, 0.8)	73	0.7 (0.6, 0.9)	0.69
IL-4, pg/mL	63	0.9 (0.7, 1.1)	73	0.8 (0.7, 1.0)	0.57
IL-5, pg/mL	63	2.0 (1.6, 2.5)	73	1.5 (1.3, 1.8)	0.07
IL-6, pg/mL	63	18.8 (12.9, 27.3)	73	19.9 (13.3, 29.9)	0.83
IL-7, pg/mL	63	8.3 (6.8, 10.3)	73	8.0 (6.8, 9.4)	0.78
IL-8, pg/mL	63	44.7 (35.7, 56.0)	73	37.9 (30.9, 46.6)	0.29
IL-10, pg/mL	63	2.0 (1.5, 2.7)	73	1.9 (1.3, 2.8)	0.91
IL-12, pg/mL	63	8.7 (6.4, 11.9)	73	6.9 (4.9, 9.7)	0.33
IL-13, pg/mL	63	2.5 (2.1, 3.1)	73	2.6 (2.1, 3.1)	0.92
IL-17, pg/mL	63	1.4 (0.9, 2.4)	73	1.3 (0.8, 2.1)	0.77
CCL2 / MCP-1, pg/mL	63	41.9 (32.9, 53.4)	73	41.8 (32.2, 54.3)	0.99
CCL3 / MIP-1α, pg/mL	63	9.6 (6.3, 14.8)	73	9.4 (6.4, 14.0)	0.94
CCL4 / MIP-1β, pg/mL	63	164 (123, 218)	73	173 (132, 226)	0.77
sCD14, ng/mL	63	4.4 (4.0, 4.9)	73	4.2 (3.7, 4.6)	0.45
sCD163, ng/mL	63	799 (700, 912)	73	1010 (890, 1146)	0.01
CRP, mg/L	58	51.2 (39.3, 66.8)	67	33.4 (25.1, 44.5)	0.04

Biomarker values are geometric mean values from log2 transformation. P-values are from linear regression.

G-CSF = granulocyte colony stimulating factor; GM-CSF = granulocyte-monocyte colony stimulating factor; IFN = interferon; TNF = tumor necrosis factor; VEGF = vascular endothelial growth factor; IL = interleukin; CCL2 = MCP-1 = monocyte chemoattractant protein-1; CCL3 = MIP1-α = Macrophage inflammatory protein-1 alpha; CCL4 = MIP-1β = Macrophage inflammatory protein-1-beta; CRP = C-reactive protein.

Supplemental Table 4a – Differences in CSF biomarkers at randomization (amphotericin day 8) between individuals with CSF White Cells <5 and ≥5 cells/µL. Individuals with CSF WCC <5/µL had significantly higher CSF GM-CSF, and significantly reduced sCD14 and sCD163 compared to individuals with CSF WCC ≥5/µL. Biomarker values are geometric mean values from log2 transformation. P-values are from linear regression of biomarker on CSF white cell count at randomization group.

CSF Biomarker	CSF White Cells < 5/µL		CSF White Cells >= 5/µL		P-value
	N	Mean (95% CI)	N	Mean (95% CI)	
G-CSF, pg/mL	47	40.0 (31.0, 51.7)	62	42.2 (33.2, 53.7)	0.77
GM-CSF, pg/mL	47	330 (294, 369)	62	219 (174, 276)	<.01
IFN-γ, pg-mL	47	27.4 (18.7, 39.9)	62	24.1 (16.6, 34.9)	0.64
TNF-α, pg/mL	47	5.9 (4.4, 7.8)	62	6.8 (5.4, 8.6)	0.43
VEGF, pg/mL	27	29.5 (18.0, 48.2)	37	25.3 (14.3, 44.9)	0.71
IL-4, pg/mL	47	0.7 (0.6, 0.9)	62	0.7 (0.6, 0.9)	0.91
IL-6, pg/mL	47	79.2 (43.7, 144)	62	138 (83.5, 229)	0.16
IL-7, pg/mL	47	3.9 (2.9, 5.3)	62	4.0 (3.1, 5.1)	0.92
IL-8, pg/mL	47	406 (291, 566)	62	477 (352, 646)	0.49
IL-10, pg/mL	47	8.5 (6.8, 10.6)	62	7.5 (6.0, 9.4)	0.47
IL-12, pg/mL	47	5.7 (3.9, 8.2)	62	6.1 (4.6, 7.9)	0.78
IL-13, pg/mL	47	14.3 (8.7, 23.5)	62	18.7 (11.9, 29.3)	0.44
IL-17, pg/mL	47	6.3 (4.0, 9.8)	62	5.1 (3.3, 7.7)	0.51
CCL2 / MCP-1, pg/mL	47	593 (382, 920)	62	455 (320, 646)	0.35
CCL3 / MIP-1α, pg/mL	6	8.7 (6.7, 11.2)	26	5.4 (3.4, 8.6)	0.36
CCL4 / MIP-1β, pg/mL	47	108.5 (91.2, 129)	62	103.5 (86.5, 124)	0.72
sCD14, ng/mL	43	524 (372, 737)	59	800 (639, 1002)	0.04
sCD163, ng/mL	43	427 (305, 599)	59	676 (505, 905)	0.05
		N (%) Detectable		N (%) Detectable	
IL-1β, pg/mL	47	23 (49%)	62	27 (44%)	0.58
IL-2, pg/mL	47	18 (38%)	62	32 (52%)	0.17
IL-5, pg/mL	47	25 (53%)	62	34 (55%)	0.87
CCL22 / MDC, pg/mL	43	27 (63%)	59	48 (81%)	0.04

G-CSF = granulocyte colony stimulating factor; GM-CSF = granulocyte-monocyte colony stimulating factor; IFN = interferon; TNF = tumor necrosis factor; VEGF = vascular endothelial growth factor; IL = interleukin; CCL2 = MCP-1 = monocyte chemoattractant protein-1; CCL4 = MIP-1β = Macrophage inflammatory protein-1-beta; CCL22 = MDC = macrophage-derived chemokine

Supplemental Table 4b – Differences in serum biomarkers at randomization (amphotericin day 8) between individuals with CSF white cell counts (WCC) <5 and ≥5 cells/μL. Individuals with CSF WCC <5/μL had significantly higher serum IL-4, IL-17 concentrations compared to individuals with CSF WCC ≥5/μL.

Serum Biomarker	CSF White Cells <5/μL		CSF White Cells >5/μL		P-value
	N	Mean (95% CI)	N	Mean (95% CI)	
G-CSF, pg/mL	61	17.5 (12.6, 24.2)	72	15.2 (11.7, 19.7)	0.51
GM-CSF, pg/mL	61	5.2 (3.6, 7.4)	72	4.2 (2.8, 6.1)	0.42
IFN-γ, pg/mL	61	56.2 (34.9, 90.3)	72	32.8 (22.7, 47.4)	0.08
TNF-α, pg/mL	61	8.4 (6.2, 11.3)	72	8.2 (6.4, 10.5)	0.91
VEGF, pg/mL	61	177 (120, 260)	72	141 (98.2, 202)	0.40
IL-1β, pg/mL	61	1.2 (0.9, 1.4)	72	0.9 (0.8, 1.1)	0.11
IL-2, pg/mL	61	2.0 (1.2, 3.3)	72	1.7 (1.1, 2.6)	0.64
IL-4, pg/mL	61	1.5 (1.2, 2.0)	72	1.1 (0.9, 1.3)	0.05
IL-5, pg/mL	61	2.0 (1.5, 2.7)	72	1.6 (1.3, 2.0)	0.20
IL-6, pg/mL	61	38.8 (24.9, 60.6)	72	32.6 (23.6, 45.1)	0.53
IL-7, pg/mL	61	9.8 (8.1, 12.0)	72	8.9 (7.7, 10.4)	0.44
IL-8, pg/mL	61	49.3 (38.4, 63.2)	72	49.8 (39.6, 62.5)	0.95
IL-10, pg/mL	61	3.0 (1.9, 4.7)	72	2.1 (1.5, 3.0)	0.24
IL-12, pg/mL	61	14.2 (10.9, 18.6)	72	10.9 (8.4, 14.1)	0.16
IL-13, pg/mL	61	2.5 (1.8, 3.3)	72	3.0 (2.3, 4.0)	0.32
IL-17, pg/mL	61	9.1 (5.4, 15.3)	72	4.4 (2.7, 7.2)	0.05
CCL2 / MCP-1, pg/mL	61	72.8 (53.2, 99.7)	72	58.1 (43.2, 78.3)	0.31
CCL4 / MIP-1β, pg/mL	61	361 (291, 449)	72	278 (227, 341)	0.09
CRP, mg/L	61	84.0 (67.3, 105)	72	91.5 (75.2, 111)	0.57

Biomarker values are geometric mean values from log2 transformation. P-values are from linear regression.

G-CSF = granulocyte colony stimulating factor; GM-CSF = granulocyte-monocyte colony stimulating factor; IFN = interferon; TNF = tumor necrosis factor; VEGF = vascular endothelial growth factor; IL = interleukin; CCL22 = MDC = macrophage-derived chemokine; CCL2 = MCP-1 = monocyte chemoattractant protein-1; CCL3 = MIP1-α = Macrophage inflammatory protein-1 alpha; CCL4 = MIP-1β = Macrophage inflammatory protein-1-beta; CRP = C-reactive protein. sCD14 and sCD163 were not measured at randomization.

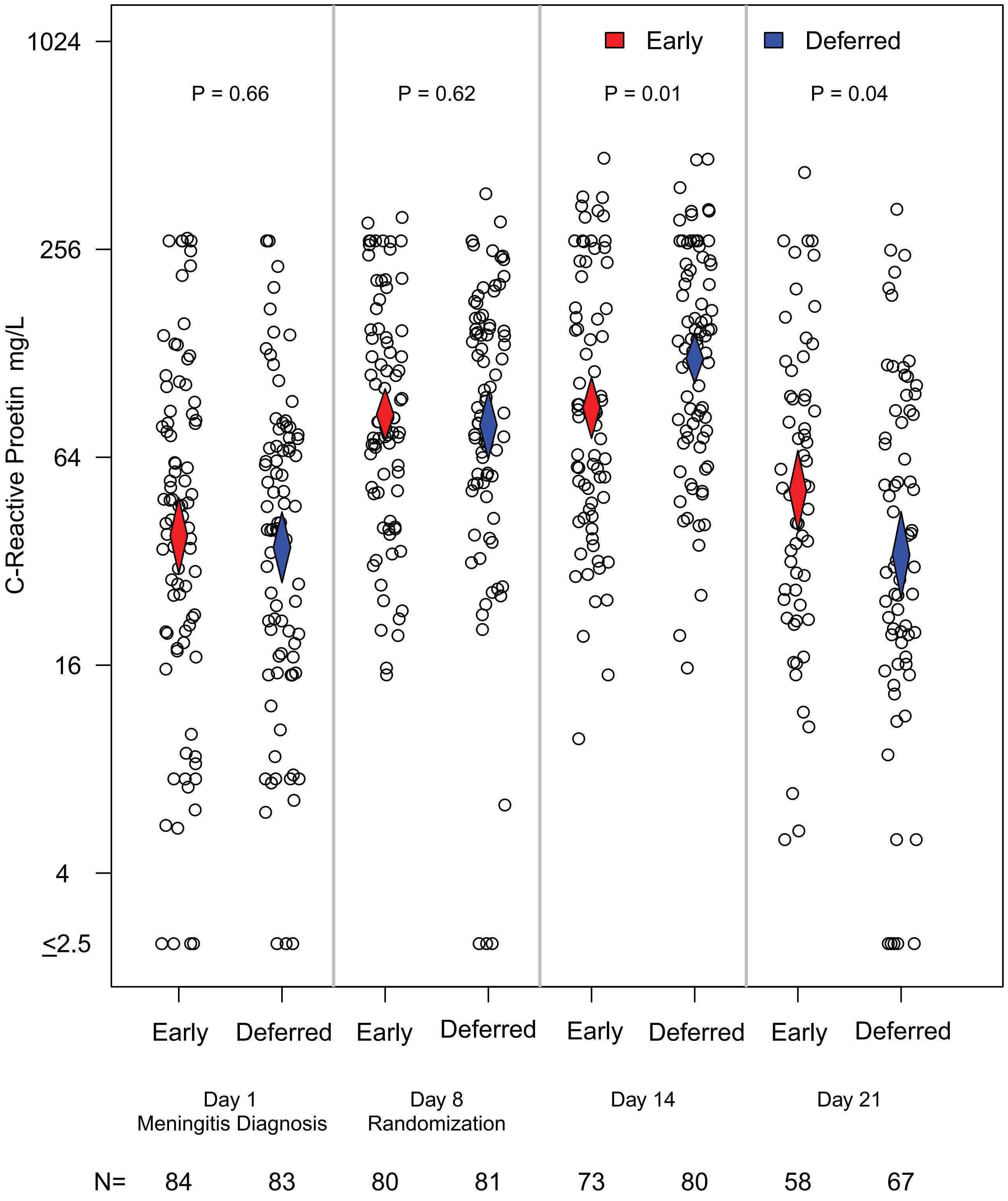

C-Reactive Proetin mg/L
1024
256
64
16
4
≤2.5
Early
Deferred
P = 0.66
P = 0.62
P = 0.01
P = 0.04
Early
Deferred
Early
Deferred
Early
Deferred
Early
Deferred
Day 1
Meningitis Diagnosis
Day 8
Randomization
Day 14
Day 21
N= 84 83 80 81 73 80 58 67
